# Anti-fibrotic effects of *Cuscuta chinensis* with *in vitro* hepatic stellate cells and a thioacetamide-induced experimental rat model

**DOI:** 10.1080/13880209.2017.1340965

**Published:** 2017-06-27

**Authors:** Jin Seoub Kim, Sushruta Koppula, Mun Jeong Yum, Gwang Mo Shin, Yun Jin Chae, Seok Min Hong, Jae Dong Lee, MinDong Song

**Affiliations:** a Department of Applied Life Science, Graduate School of Konkuk University, Chungju-si, South Korea;; b Department of Infectious Disease, Asan Institute for Life Science, Asan Medical Center, University of Ulsan College of Medicine, Seoul, South Korea;; c Department of Biotechnology, College of Biomedical and Health Sciences, Konkuk University, Chungju-si, South Korea;; d R&D Center Korean Drug Co., Ltd, Seoul, South Korea;; e Medrim Corp, Chungju-si, South Korea;; f Department of Internal Medicine, School of Medicine, Konkuk University, Chungju-si, South Korea

**Keywords:** Glutathione, hydroxyproline, apoptosis, silymarin, aspartate, alanine

## Abstract

**Context:***Cuscuta chinensis* Lam. (Convolvulaceae) has been used as a traditional herbal remedy for treating liver and kidney disorders.

**Objective:** Anti-fibrotic effects of *C. chinensis* extract (CCE) in cellular and experimental animal models were investigated.

**Materials and methods:** HSC-T6 cell viability, cell cycle and apoptosis were analysed using MTT assay, flow cytometry and Annexin V-FITC/PI staining techniques. Thioacetamide (TAA)-induced fibrosis model was established using Sprague Dawley rats (*n* = 10). Control, TAA, CCE 10 (TAA with CCE 10 mg/kg), CCE 100 (TAA with CCE 100 mg/kg) and silymarin (TAA with silymarin 50 mg/kg). Fibrosis was induced by TAA (200 mg/kg, *i.p.*) twice per week for 13 weeks. CCE and silymarin were administered orally two times per week from the 7th to 13th week. Fibrotic related gene expression (α-SMA, Col1α1 and TGF-β1) was measured by RT-PCR. Serum biomarkers, glutathione (GSH) and hydroxyproline were estimated by spectrophotometer using commercial kits.

**Results:** CCE (0.05 and 0.1 mg/mL) and silymarin (0.05 mg/mL) treatment significantly (*p* < 0.01 and *p* < 0.001) induced apoptosis (11.56%, 17.52% for CCE; 16.50% for silymarin, respectively) in activated HSC-T6 cells, compared with control group (7.26%). Further, rat primary HSCs showed changes in morphology with CCE 0.1 mg/mL treatment. In *in vivo* studies, CCE (10 and 100 mg/kg) treatment ameliorated the TAA-induced altered levels of serum biomarkers, fibrotic related gene expression, GSH, hydroxyproline significantly (*p* < 0.05–0.001) and rescued the histopathological changes.

**Conclusions:** CCE can be developed as a potential agent in the treatment of hepatofibrosis.

## Introduction

*Cuscuta chinensis* Lam. (Convolvulaceae), originally from China, has been used as an herbal medicine in several Asian countries for centuries. In Traditional Chinese Medicine (TCM), the semen of *C. chinensis* has been used as a tonic and aphrodisiac to improve sexual potency, prevent abortion and to enhance liver and kidney conditions (Donnapee et al. 2014). Pharmacologically, *C. chinensis* possess neuroprotective (Zhen et al. 2006), hepatoprotective, antioxidant (Yen et al. 2007), osteoblastogenic (Yang et al. 2009), genoprotective activities (Szeto et al. 2011) and improve renal function in experimental rats (Shin et al. 2011). Although *C. chinensis* showed a broad range of biological activities, there is no scientific evidence regarding the anti-fibrotic effects.

Hepatofibrosis results from chronic damage to the liver in conjunction with the accumulation of extracellular matrix (ECM) proteins, which is a characteristic of most types of chronic liver diseases (Friedman 2003). Hepatic fibrosis was historically thought to be a passive and irreversible process due to the collapse of the hepatic parenchyma and its substitution with a collagen-rich tissue (Schaffner and Klion 1968; Popper and Uenfriend 1970). Hepatic fibrosis is associated with activation of hepatic stellate cells (HSCs), the major source of the ECM proteins and is also caused by frequent hepatic injury with sustained inflammation in liver tissue and organ failure (Bruck et al. 2001; Henderson and Iredale 2007).

HSCs are considered as key participants in liver fibrosis development which is central process of fibrosis as the major source of fibrillary and non-fibrillar matrix protein (Iredale et al. 1998; Abramovitch et al. 2011). HSCs are usually quiescent cells, but in response to liver injury they undergo an activation process in which they become highly proliferative and synthesize a fibrotic matrix rich in type I collagen (Reeves and Friedman 2002). The phenotypic changes seen in activated HSCs often characterized as ‘myofibroblastic activation’ lead to excessive deposition of ECM and disrupt the normal architecture of the liver causing liver fibrosis, liver cirrhosis and liver cancer (Friedman 2003; Tsukada et al. 2006; Yoon et al. 2016). Therefore, it is important to induce the apoptosis of HSCs or prevent the secretion of the ECM by HSCs (Lee et al. 2014). Thus, in the present study, we investigated the antifibrotic effects of *C. chinensis* extracts (CCEs) in an *in vitro* system using HSC-T6 cells and an *in vivo* system using thioacetamide (TAA)-induced liver fibrosis rat model.

## Materials and methods

### Materials

Silymarin, TAA, hydroxyproline, *p*-dimethylaminobenzaldehyde, 1,1,3,3-tetraethoxypropane, chloramines-T, 5,5-dithiobis-2-nitrobenzoic acid (DTNB), glutathione (GSH), β-nicotinamide adenine dinucleotide phosphate, reduced form (β-NADPH) were purchased from Sigma (St. Louis, MO); perchloric acid was obtained from GFS Chemical Co. (Columbus, OH). All other reagents used in this study were of highest grade available commercially.

### Plant material and extraction

*C. chinensis*, collected during May 2016, was purchased from Jecheon Chinese Medicinal Plant Co., Jecheon, South Korea and was authenticated by Prof. Jong-Bo Kim, a taxonomist at Konkuk University, South Korea, based on its microscopic and macroscopic characteristics. A voucher specimen (CC-KU2016) was kept in our department herbarium for future reference. For extraction, the dried semen of *C. chinensis* (300 g) was ground to a fine powder and extracted with 1 L ethanol (95%) using Soxhlet’s extraction technique for three days at room temperature. The extract was then concentrated in a vacuum under reduced pressure and lyophilized. The final yield of the lyophilized CCE was 9.5% (w/w) and was stored at 4 °C. The lyophilized powder of CCE was dissolved in 10% dimethyl sulphoxide (DMSO) and then filtered through a 0.22 μM syringe filter and stored as stock until use for each experiment. The final concentration of DMSO used for the study was not more than 0.1%.

### Cell lines and culture

An immortalized rat’s hepatic stellate cell lines (HSC-T6) were generously provided by Prof. Chang-Gue Son (Korean Hospital of Daejeon University, South Korea). HSC-T6 were cultured in Dulbecco’s modified Eagle’s medium (DMEM) supplemented with 5% FBS, 1% antibiotic–antimycotic in a humidified atmosphere of 5% CO_2_ at 37 °C. Chang liver cell line was purchased from ATCC (Manassas, VA). Chang liver cell line was used as a normal human cell line derived from normal liver tissue. The cells were cultured in DMEM (GIBCO, Carlsbad, CA) supplemented with 10% foetal bovine serum (FBS, GIBCO, Carlsbad, CA), 1% antibiotic–antimycotic (Invitrogen, Carlsbad, CA) in a humidified atmosphere of 5% CO_2_ at 37 °C. For activation, HSC-T6 cells were serum starved before treatment with CCE.

### Primary HSCs isolation and culture

HSCs were isolated from 7-week-old male Sprague Dawley (SD) rats by *in situ* with pronase, collagenase, DNase perfusion and single-step Histogenz gradient as previously reported (Knook et al. 1982; Hendriks et al. 1985). Isolated HSCs were cultured in low glucose DMEM (GIBCO, Carlsbad, CA) containing 10% FBS (GIBCO, Carlsbad, CA) and 1% antibiotic–antimycotic (Invitrogen, Carlsbad, CA) on uncoated plastic maintained in a humidified atmosphere of 5% CO_2_ at 37 °C and these activated HSCs were used in the experiments. The growth medium was changed on a daily basis for seven days.

### Cell viability assay

Cell viability assays were evaluated by the 3-(4,5-demethylthiazol-2yl)-2,5-diphenyl-2H-tetrazolium bromide (MTT) method. In a 96-well plate, Chang cell (7 × 10^5^ cell/well), HSC-T6 (6 × 10^5^ cells/well) were cultured in DMEM medium supplemented as described previously. Sample material was evaluated at various concentrations (0, 0.01, 0.05, 0.1, 0.5 and 1.0 mg/mL) for 24 h at 37 °C in an atmosphere of 5% CO_2_ and 95% humidity. The cells were then incubated with 0.1 mg/mL MTT (SIGMA, St. Louis, MO) for 3 h, and the reaction was interrupted by addition of dimethyl sulphoxide (DMSO, JUNSEL, Tokyo, Japan). An ELISA reader was used to obtain the results at 540 nM. The viabilities of the control cells were used as the control values at 100%.

### Cell cycle analysis

HSC-T6 cells (15 × 10^5^ cells/well) were cultivated in DMEM medium containing 10% FBS (GIBCO, Carlsbad, CA) and 1% antibiotic–antimycotic (GIBCO, Carlsbad, CA) maintained in a humidified atmosphere of 5% CO_2_ at 37 °C. Growth medium was changed on a daily basis for seven days. Sample materials were evaluated at CCE 0.05 and 0.1 mg/mL concentrations for 24 h at 37 °C in an atmosphere of 5% CO_2_ and 95% humidity. After 24 h, the cells were washed with PBS twice, suspended in 1 mL cold PI solution (50 μg/mL PI and 100 μg/mL RNase A). Then, the cells were incubated on ice for 30 min in the dark and then analysed with a flow cytometer.

### Apoptosis analysis

Apoptosis was determined by Annexin V-FITC and PI (FICS Annexin V apoptosis Detection Kit I, BD Biosciences, Franklin Lakes, NJ). The processes of detection were carried out according to manufacturer’s instruction. Data analysis was performed with CellQuest software (Beckton Dickinson, Franklin Lakes, NJ), which allowed assessing of specific population only. Individualization by gates was done according to size, granularity and fluorescent parameters. Both early apoptotic (Annexin V^+^ and PI^−^) and late apoptotic (Annexin V^+^ and PI^−^) cells were included in cell death determinations.

### Animals and experiment design

Fifty specific-pathogen-free SD male rats (six-weeks old, 190–210 g) were purchased from a commercial animal breeder (Orient Bio, Seoul, Korea). Animals were housed in conventional cages under control conditions of temperature (23 ± 3 °C), relative humidity (50 ± 20%) and 12 h light/dark cycle. After 1 week of acclimation, the rats were divided randomly into five groups of 10 animals each: Normal, TAA (TAA only), CCE 10 (TAA with CCE 10 mg/kg), CCE 100 (TAA with CCE 100 mg/kg) and positive control silymarin group (TAA with 50 mg/kg silymarin). Liver fibrosis was induced using a previously described procedure with slight modifications (Wallace et al. 2015). Briefly, TAA (200 mg/kg) was administered intraperitoneally (*i.p.*) twice a week for 13 weeks to four groups except normal group (injected normal saline, *i.p.*). CCE (10 or 100 mg/kg), silymarin (50 mg/kg) or distilled water was given by gastric gavage six times per week from the 7th week to the 13th week. Body weight was recorded once a week. After last CCE or silymarin administration, animals were fasted for 18 h, and then blood was collected from cardiac puncture under CO_2_ anaesthesia. A portion of liver tissue stored at −80 °C separately was used for hydroxyproline, GSH, protein expression determination. Liver tissue fixed in Bouin’s solution was processed for histomorphological findings. A small portion of liver tissue fixed in RNAlater solution was stored at −80 °C for gene expression studies. All animal experiments were approved by the Committee of Laboratory Animals according to the institutional guidelines of Konkuk University, South Korea (IACUC No. KU15017).

### Serum biochemical analysis

Blood was collected through cardiac puncture under CO_2_ anaesthesia on the final day of the experiments. Serum was separated using centrifugation (3000×*g*, 15 min). Following the blood clotting, the serum levels of aspartate transaminase (AST) and alanine transaminase (ALT) were determined using a GOT-GTP assay kit (Asan Pharmaceutical, Anseong-si, Korea).

### Estimation of total GSH content

Total GSH was determined according to the method of Evans and Ellman (1959). Briefly, duplicate 50 μL aliquots of the samples (or GSH standard) were combined with 80 μL of a previously prepared DTNB. NADPH mixture (10 μL 4 mM DTMB and 70 μL 0.3 mM NADPH) is taken in a 96-well plate. Finally, 20 μL (0.06 U) of GSH reductase solution was added to each well and the absorbance was ensured at 405 nM after 5 min.

### Determination of hydroxyproline in liver tissues

Hydroxyproline determination was performed using a light modification in the previous method (Takayama et al. 2003). Briefly, liver tissues (156 mg) stored at −70 °C were homogenized in 1 mL of 6 N HCl and incubated overnight at 100 °C. After passage of the acid hydrolysates through filter paper (Toyo Roshi Kaisha, Tokyo, Japan), 50 μL samples or hydroxyproline standards in 6 N HSL were air-dried. The dried samples were dissolved in methanol (50 μL), and then 1.2 mL of 50% isopropanol and 200 μL of chloramine-T solution were added to each followed by incubation at room temperature for 10 min. Ehrlich’s solution (1.3 mL) was added, and the samples were incubated at 50 °C for 90 min. The optical density of the reaction product was read at 558 nM using a spectrophotometer (Tecan, Morrisville, NC). A standard curve was constructed using serial dilutions of 0.1 mg/mL hydroxyproline solution.

### Histopathology of liver tissue

Bouin’s solution fixed liver tissues were embedded in paraffin and cut into 5 μM thick section for histomorphological examination. After drying, liver tissue section slides were stained with haematoxylin and eosin (H&E) and Masson’s trichrome. For semiquantitative analysis of collagen expression, the blue-stained areas in Masson’s trichrome stained sections were measured on an image analyser (ImageJ, NIH, Bethesda, MD).

### Real time-polymerase chain reaction (qRT-PCR)

Total RNA was extracted from liver tissue samples and HSC-T6 cells using TRIzol reagent (QIAGEN, Valencia, CA). Total RNA (2 μg) was used in a 20 μL reaction cDNA synthesis using a high-capacity cDNA reverse transcription kit (Applied Biosystems, Foster City, CA). The primers for α-smooth muscle actin (α-SMA), collagen type 1 α 1 (Col1α1), transforming growth factor β 1 (TGF-β1) and β-actin were as follows α-SMA (forward sequence, 5′-AACACGGCATCATCACCAACT-3′; reverse sequence, 5′-TTTCTCCCGGTTGGCCTTA-3′), Col1α1 (forward sequence, 5′-CCCAGCGGTGGTTATGACTT-3′; reverse sequence, 5′-GCTGCGGATGTTCTCAATCTG-3′), TGF-β1 (forward sequence, 5′-AGGAGACGGAATACAGGGCTTT-3′; reverse sequence, 5′-AGC AGGAAGGGTCGGTTCAT-3′), β-actin (forward sequence, 5′-CTAAGGCCAACCGTGAAAAGAT-3′; reverse sequence, 5′-GACCAGAGGCATACAGGGACAA-3′). The processes of reactions were carried out according to manufacturer’s instructions. For analysis of data, the gene expression levels were normalized with β-actin as a reference gene.

### Statistical analysis

The results are expressed as the mean ± standard error of the mean (S.E.M., *n* = 10). Statistical analysis was carried out using Student’s *t*-test using Graph Pad Prism software version 4.00 (Graph Pad Software Inc., San Diego, CA). Differences between groups were analysed by one-way analysis of variance (ANOVA). The values of *p* < 0.05 were considered as statistically significant.

## Results

### Cell viability assay and primary HSC morphology

**Figure 1.**
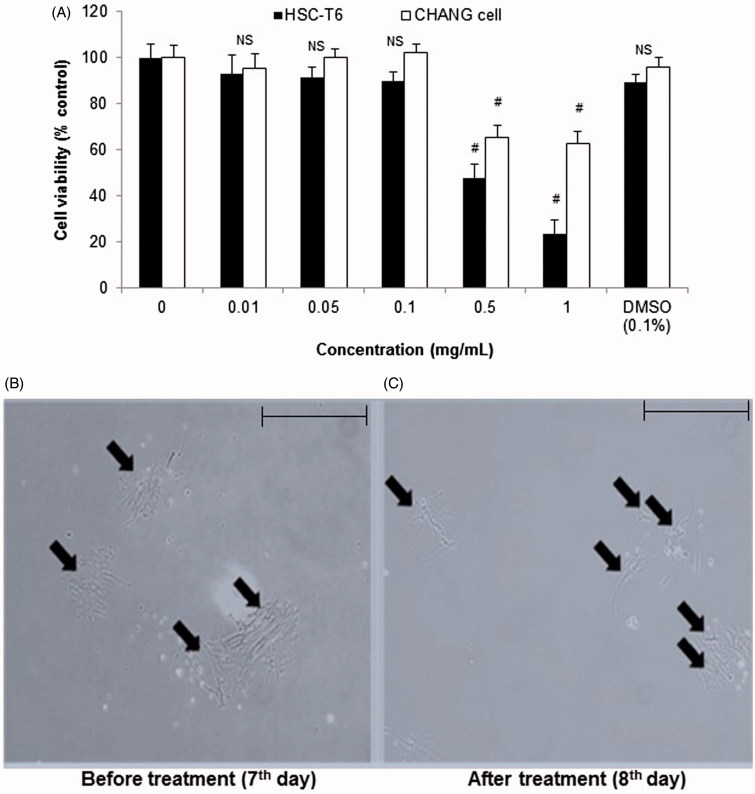
Cell viability assay on Chang liver/HSC-T6 cells and morphological changes in primary HSCs on treatment with CCE. (A) Chang liver and HSC-T6 cells were incubated with CCE at indicated concentrations for 24 h and the cell viability was determined by MTT assay. (B) Primary HSCs were cultivated for 1 week and exposed to the CCE 0.1 mg/mL for 24 h (C). Pictures were taken after 24 h treatment with CCE. Magnification was 100×. Arrows indicate HSCs. The data are expressed as means ± S.E.M. (*n* = 10), using one-way analysis of variance (ANOVA) followed by Student’s *t*-test. #*p* < 0.05, compared with control group. NS: not significant compared with control group.

As shown in Figure 1(A), CCE was treated at various concentrations (0.01, 0.05, 0.1, 0.5, and 1.0 mg/mL) in Chang liver cell and HSC-T6 cells. CCE treated at indicated concentrations (0.05 and 0.1 mg/mL) did not exhibit any significant changes in the overall cell viability or produce toxicity in Chang liver cells and HSC-T6 cells. However, concentrations greater than 0.5 mg/mL showed significant effect on cell viability. Further the solvent, DMSO (0.1%) alone used for dissolving the CCE also did not show significant toxicity. Therefore, all *in vitro* experiments were performed with CCE 0.05 and/or 0.1 mg/mL as the concentrations were considered nontoxic and effective (Figure 1(A)).

As shown in Figure 1(B), untreated activated HSCs showed normal morphology (7th day). CCE (0.1 mg/mL) treatment for 24 h on eight day-cultured primary HSCs showed changes in cell morphology such as shrinking collagen fibre and cell degradation (Figure 1(C)). This indicated that CCE treatment influenced the morphology of the cultured activation HSCs by decreasing the number of viable HSCs and stretched fibres when compared with the non-treated activated HSCs after 24 h.

### Cell cycle analysis

**Figure 2.**
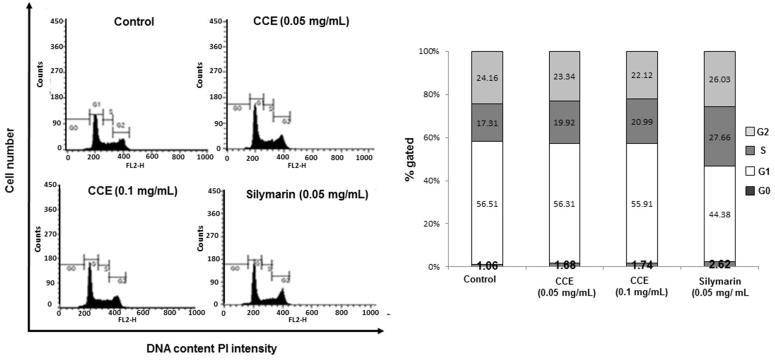
Effect of CCE on the cell cycle in HSC-T6 cells. DNA contents in different phases of the cell cycle were measured using propidium iodide by flow cytometry. The cell cycle distribution and the percentage of the cell cycle distribution were represented by histogram (left) and graphs (right), respectively.

Flow cytometric analysis of CCE (0.05 and 0.1 mg/mL) treated HSC-T6 cells showed 1.74 and 2.62%, respectively, in the sub-G1 phase compared with non-treated cells showing a distribution of 1.06%. Silymarin was used as a positive control, and showed 1.68% of the cells in the sub-G1 phase. These results indicated that CCE treatment has mild effects including the induction of apoptosis in HSC-T6 cells (Figure 2).

### Apoptosis analysis on HSC-T6 cells

**Figure 3.**
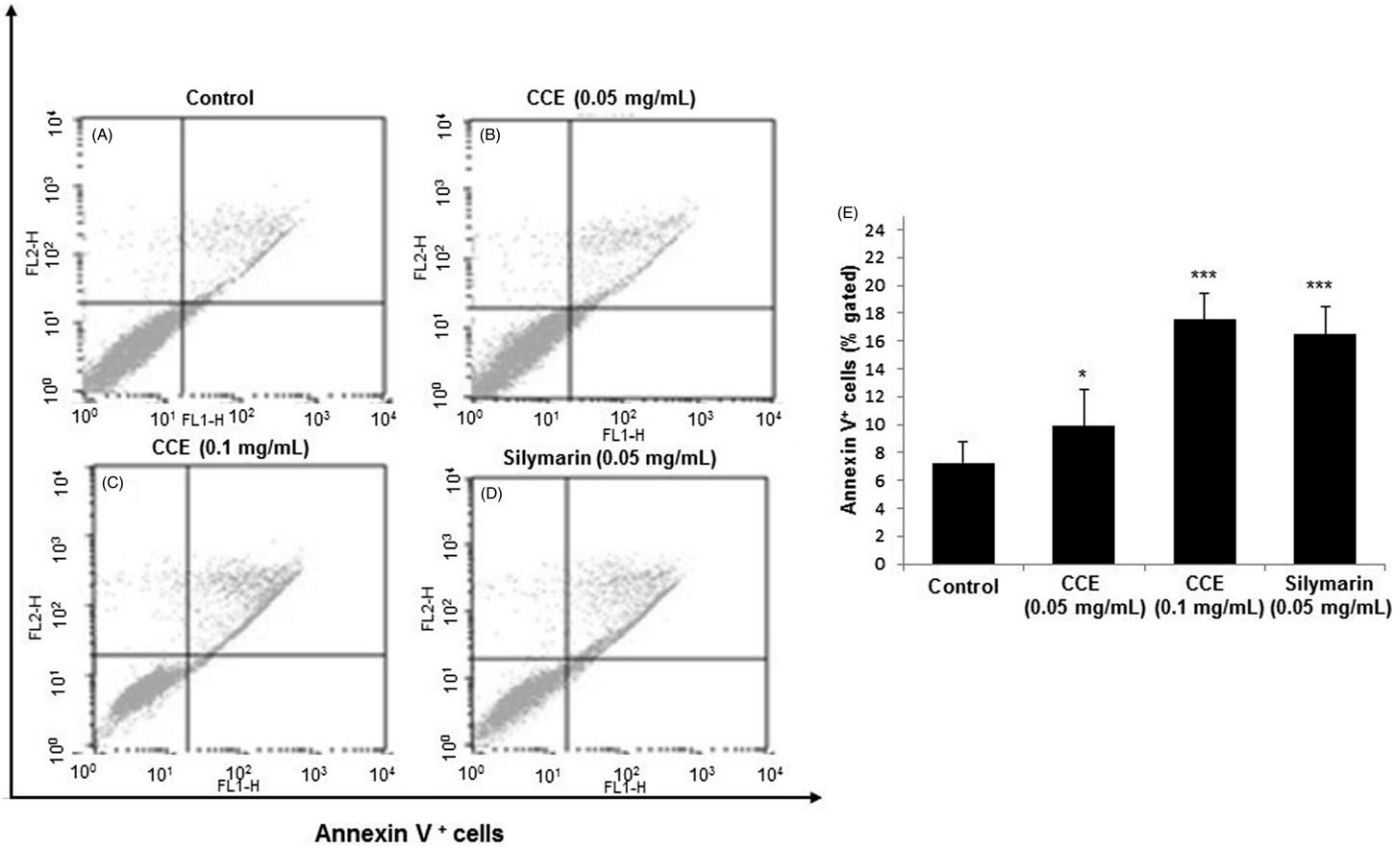
Effect of CCE on apoptosis in activated HSC-T6 cells. (A) Control cells. Flow cytometric data indicate apoptosis in HSC-T6 cells after incubation with CCE 0.05 mg/mL (B), CCE 0.1 mg/mL (C) and silymarin 0.05 mg/mL (D) for 24 h. E: Data showed the apoptotic (Annexin V^+^ and PI^−^) and late apoptotic (Annexin V^+^ and PI^+^) cells. Data are represented as mean ± S.E.M. (*n* = 10) using one-way analysis of variance (ANOVA) followed by Student’s *t*-test. *p < 0.05 and ***p < 0.001, as compared with control group.

As shown in Figure 3, HSC-T6 cells were treated with silymarin (0.05 mg/mL) and CCE (0.05 and 0.1 mg/mL) for 24 h. The percentage of cells undergoing apoptotic cell death increased from 7.26 ± 1.54 (control group) to 16.50 ± 2.65 (silymarin), 11.16 ± 1.89% (CCE 0.05 mg/mL) and 17.53 ± 2.01% (CCE 0.1 mg/mL) groups, respectively, for 24 h (Figure 3(A–D)). CCE 0.1 mg/mL and silymarin exhibited about two times more potency than Annexin V positive control cells (*p* < 0.001). CCE 0.05 mg/mL also showed significant effects (*p* < 0.05) in inducing apoptosis (Figure 3(E)). These results indicated that CCE induced the apoptosis on HSC-T6 cells.

### Serum biochemical analysis

**Figure 4.**
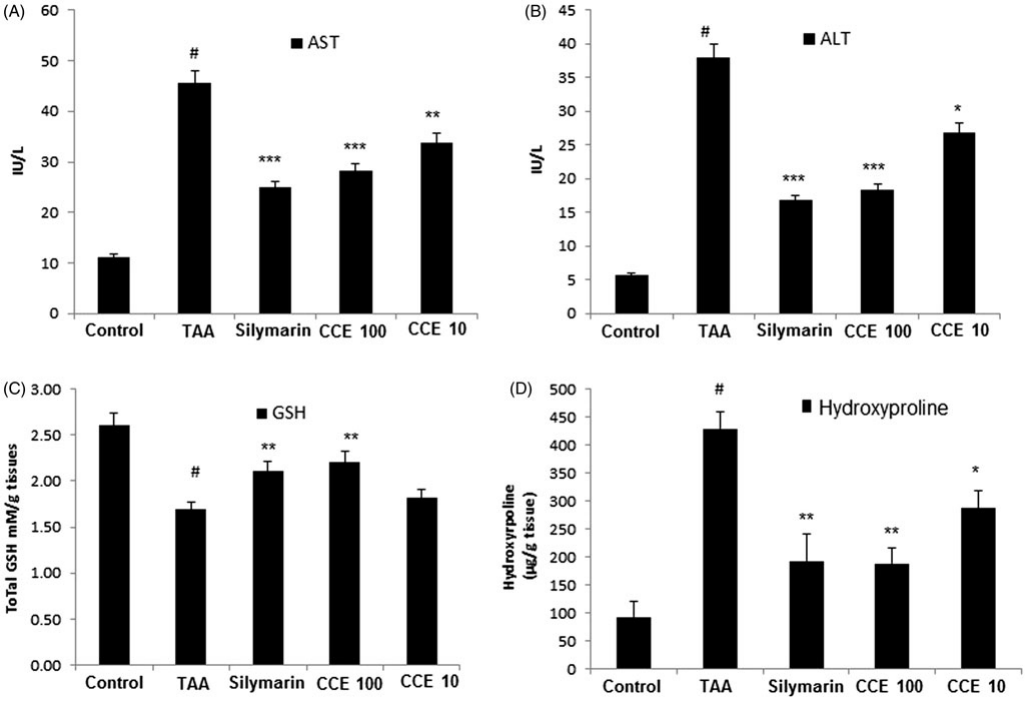
Effect of CCE on AST/ALT levels, total glutathione (GSH) contents and hydroxyproline levels in TAA-induced liver fibrosis rats. (A) HSC-T6 cells were incubated with CCE and silymarin for 24 h. Levels of AST (A) and ALT (B) in serum were measured using spectrophotometry. Total GSH contents (C) and hydroxyproline levels (D) in liver tissues were measured using spectrophotometry. TAA (200 mg/kg): thioacetamide-induced liver fibrosis rats, silymarin (50 mg/kg): positive control rats, CCE 100: CCE 100 mg/kg treated rats, CCE 10: CCE 10 mg/kg treated rats. The data are expressed as means ± S.E.M. (*n* = 10) using one-way analysis of variance (ANOVA) followed by Student’s *t*-test. #*p* < 0.05 as compared with control group, *p < 0.05, **p < 0.01, ***p < 0.001 as compared with TAA group.

As shown in Figure 4(A,B), TAA-induced group significantly (*p* < 0.001) increased the AST and ALT serum levels. However, the levels of AST and ALT were significantly (*p* < 0.5∼*p* < 0.001) decreased by CCE (10 and 100 mg/kg) treatment compared to TAA group. Especially, the AST value of CCE100 group was decreased about half compared to TAA group. Silymarin treatment also showed a positive trend with significant effect (*p* < 0.001).

### Total glutathione contents in TAA-induced liver tissues

TAA treatment significantly (*p* < 0.001) reduced the total GSH contents in the liver tissues compared to normal group. CCE treated groups restored the TAA-induced decrease in total GSH significantly (*p* < 0.01). CCE 100 group showed superior effect in restoring the total GSH level compared with silymarin treated group (Figure 4(C)).

### Determination of hydroxyproline in TAA-induced liver tissues

The hydroxyproline levels of TAA group significantly (*p* < 0.001) increased compared to control group in TAA-induced liver tissues. CCE treatment groups at both doses (10 and 100 mg/kg) attenuated this increase significantly (*p* < 0.05 and *p* < 0.01) compared with TAA group. CCE 100 group decreased hydroxyproline level same as to standard silymarin group (Figure 4(D)).

### Histopathology of liver tissues

**Figure 5.**
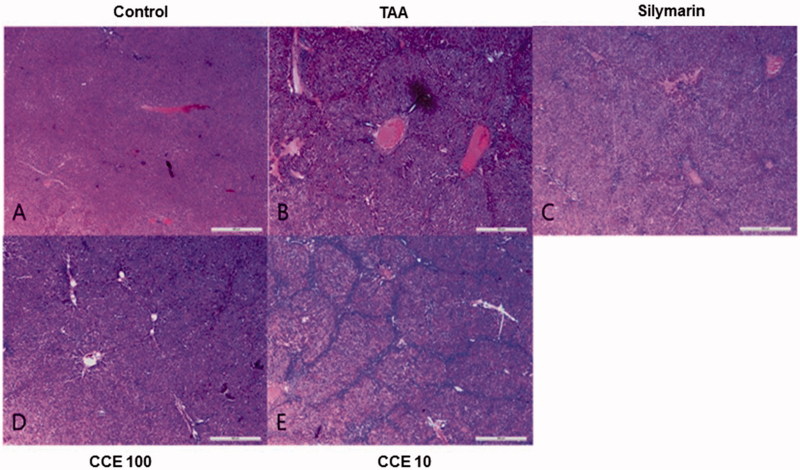
Haematoxylin and eosin (H&E) stain of liver tissues. At the end of the experiment, all of the animals were sacrificed and livers were fixed in Bouin’s solution. After staining with H&E, liver sections were taken under light microscopy. Control (A), TAA (200 mg/kg): TAA-induced liver fibrosis rats (B), silymarin (50 mg/kg): positive control rats (C), CCE 100: CCE 100 mg/kg treated rats (D), CCE 10: CCE 10 mg/kg treated rats (E). Scale bar =200 μM.

In the liver sections, control group showed normal morphology (Figure 5(A)). TAA treatment showed abnormal liver pattern with formation of numerous nodules (Figure 5(B)). However, CCE 100 and silymarin treatment markedly attenuated these changes (Figure 5(C,D)). No obvious changes were observed in CCE 10 compared with TAA-induced group (Figure 5(E)).

**Figure 6.**
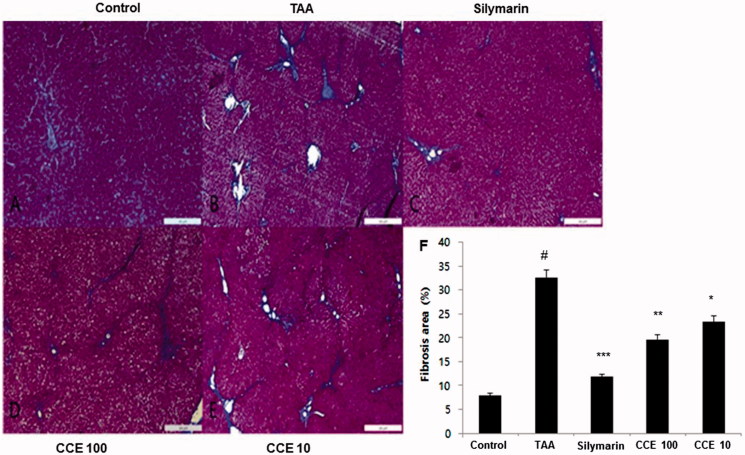
Masson’s trichrome staining of liver tissues. This stain was performed like H&E staining. Control (A), TAA (200 mg/kg): TAA-induced liver fibrosis rats (B), silymarin (50 mg/mL) (C), CCE 100 (D), CCE 10 (E) and fibrosis area plot (F). Scale bar = 200 μM. Quantification was done using ImageJ. Values are represented as mean ± S.E.M. (*n* = 10) using one-way analysis of variance (ANOVA) followed by Student’s *t*-test. #*p* < 0.05 as compared with control group, *p < 0.05, **p < 0.01 and ***p < 0.001 as compared with TAA group. TAA: thioacetamide-induced liver fibrosis rats, silymarin: positive control rats, CCE 100: CCE 100 mg/kg treated rats and CCE 10: CCE 10 mg/kg treated rats.

Masson’s trichrome showed severe collagen accumulation (blue staining part) in the TAA group when compared with control group (Figure 6(A,B)), while the silymarin and CCE 100 group remarkably protected against collagen accumulation (Figure 6(C,D)). No remarkable changes were observed in CCE 10 group (Figure 6(E)). Fibrosis percentage area revealed significant damage in TAA treated group compared with control group (*p* < 0.001). However, CCE at both concentrations ameliorated these changes significantly (*p* < 0.05 for CCE 10 and *p* < 0.01 for CCE 100 group). Silymarin exhibited significant attenuating affect (*p* < 0.001) when compared with TAA treated group (Figure 6(F)).

### Liver fibrosis related gene analysis in TAA-induced tissues

**Figure 7.**
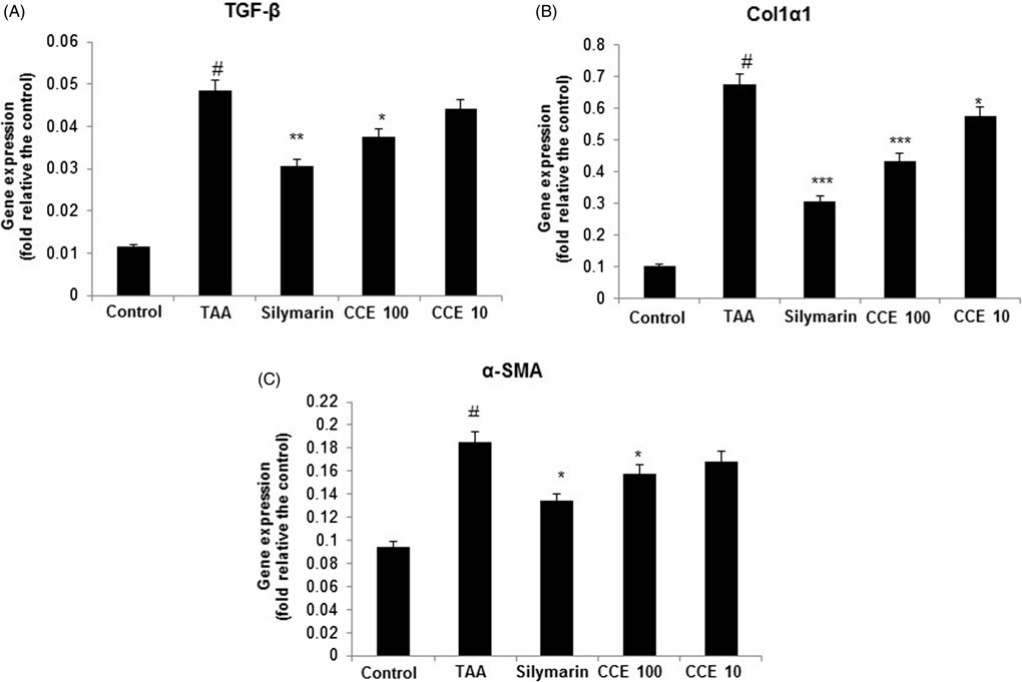
Fibrosis related gene expression analysis in the liver tissues. Fibrosis related gene expressions of liver tissue were determined by real-time-polymerase chain reaction (RT-PCR). A: TGF-β, B: Col1α1 and C: α-SMA. The results are expressed as normalized fold values relative to the control. Values are represented as mean ± S.E.M. (*n* = 10) using one-way analysis of variance (ANOVA) followed by Student’s *t*-test. #*p* < 0.05 compared with control group. *p < 0.05, **p < 0.01 and ***p < 0.001 as compared to TAA group. TAA (200 mg/kg): thioacetamide-induced liver fibrosis rats, silymarin (50 mg/kg): positive control rats, CCE 100: CCE 100 mg/kg treated rats and CCE 10: CCE 10 mg/kg treated rats.

As shown in Figure 7, TAA treatment increased the gene expression of TGF-β, Col1α1, and α-SMA significantly (*p* < 0.001). However, CCE at both concentrations and silymarin decreased the gene expression of TGF-β, Col1α1 and α-SMA (Figure 7(A–C)). Silymarin treated group exhibited superior effects when compared with CCE treated group in downregulating the TGF-β expression while showed similar effects when compared with CCE 100 group in inhibiting Col1α1, and α-SMA expression.

## Discussion

Despite considerable medical advances, liver disorders, including hepatofibrosis, remain clinically elusive with no satisfactory treatment and cure. It was well documented TCMs are currently the world’s most effective treatment for various liver disorders including cirrhosis, fibrosis and hepatitis (Dwivedi et al. 1991; Chen et al. 2010). In the present study, the Chinese traditional herb, *C. chinensis* was investigated for treating hepatofibrosis in cellular and experimental fibrotic rat model.

It is well known that HSCs activation and over expression is the key initial event in the pathogenesis of hepatofibrosis (Wang et al. 2000). Activated HSCs were also responsible for secreting collagen scar tissue, which can lead to liver cirrhosis (Morigi et al. 2004). When round-shape quiescent HSCs undergo activation by liver damage, the production of ECM is increased, and their shape changes resembling myofibroblasts (Kisseleva and Brenner 2008). Further, activated HSCs are characterized by high density of collagen around scar cell and proliferation, contractility with increased ECM productions. Thus, morphological changes in the lipid droplets that decrease stretching fibres mean the inhibition of activated HSCs. Our data suggested that CCE treatment significantly inhibited the activation and altered the morphology of HSCs.

HSCs apoptosis plays a critical role in the spontaneous recovery from fibrosis (Elsharkawy et al. 2005; Henderson and Iredale 2007). Reduced formation of procollagen and increased ECM degradation via HSCs apoptosis may benefit in steady recovery from chronic liver fibrosis. In the present study, CCE induced apoptosis in HSC-T6 cells analysed by Annexin V and PI staining technique. Further, CCE (0.1 mg/mL) downregulated the gene expression of TGF-β, α-SMA and Col1α1, selective markers of HSCs activation *in vitro.*

TAA is a carcinogen which is known to produce marked hepatotoxicity in experimental animals (Müller et al. 1988; Wallace et al. 2015). One of the major changes in TAA-induced liver damage is the altered liver enzymes such as AST and ALT which are secreted into the blood. These are the most commonly used markers of hepatocyte injury (Johnston 1999). The levels of these markers can give a general indication of whether a disorder is acute or chronic and whether it is intra- or extra-hepatic liver damage (McClatchey 2002). In the present study, the TTA-induced increase in the levels of AST and ALT enzymes was significantly reduced by CCE treatment indicating that CCE might possess beneficial property for treating hepatic injury and fibrosis.

Further, TAA-induced liver damage also produces reactive oxygen species (ROS) leading to alteration of liver biological function marker, GSH which plays a major role as a reductant in oxidation-reduction processes (Carlberg and Mannervik 1985). Therefore, restoring GSH content might be helpful in TAA-induced oxidative liver damage. In the present study, the decreased levels of GSH in TAA-induced rats were restored with CCE treatment significantly (*p* < 0.01). Hydroxyproline is a post-translational modification product of proline hydroxylation catalysed by an enzyme polyhydroxylase which is the main factor in collagen stabilization (Krane 2008; Palfi and Perczel 2008). Therefore, we determined hydroxyproline levels in liver tissues to confirm whether CCE would decrease hydroxyproline levels in TAA-induced liver tissues. TAA-induced significant increase in the hydroxyproline levels and CCE at both concentrations significantly ameliorated this increase in rat liver tissues.

TGF-β has multiple profibrogenic but also anti-inflammatory and immunosuppressive effects. The balance of these actions is required for maintaining tissue homeostasis and an aberrant expression of TGF-β is involved in a number of disease processes particularly in liver disorders (Gressner et al. 2002). TGF-β is produced by Kupffer cells and HSCs upregulate the transcription of the collagen genes (Col1α1 and Col1α2), which are observed in damaged liver and highly expressed in activated HSCs from cirrhotic liver (Jakowlew et al. 1991). Since the activated HSCs could promote the production of collagen, we also tested the effect of CCE on the expression of Col1α1 gene. An actin isoform, α-SMA is a specific marker for smooth muscle cell differentiation. Therefore, α-SMA expression has been used to identify activated HSCs that show a myofibroblastic phenotype (Carpino et al. 2005). In this report, we demonstrated that CCE significantly suppressed the TAA-induced increase in the expression of TGF-β, col1α1 and α-SMA in rat liver tissues. These results indicate that CCE exerts its antifibrotic action by inhibiting HSCs proliferation and activation.

Silymarin, the positive control used in this study, is well known for its beneficial role in liver disorders based on its antioxidant effects (Saller et al. 2001; Wu et al. 2009). In agreement, in the present study, silymarin protected TAA-induced hepatofibrosis both *in vitro* (0.5 mg/mL) and *in vivo* (50 mg/mL), however, the effects were inferior when compared with CCE at higher concentrations (0.1 mg/mL treated group *in vitro* and CCE 100 mg/kg *in vivo*).

Earlier reports revealed that antioxidants, such as flavonoids and glycosides (Kawada et al. 1998), can effectively inhibit the proliferation of HSCs. The active constituents of *C. chinensis* include flavonoids, alkaloids, saccharides, lignan and resin glycosides (Yahara et al. 1994; Miyahara et al. 1996; Du et al. 1998; Ye et al. 2002). Some of the compounds isolated from *C. chinensis* have been suggested to be responsible for the various pharmacological activities including anti-oxidant activity (Chen et al. 2004) and anti-inflammatory activities (Liao et al. 2013). The compounds present in the CCE might act individually or synergistically in delivering such potent anti-hepatofibrotic effects. Therefore, isolation of single active constituents present in CCE exploring the detailed mechanism is quite necessary.

## Conclusions

CCE showed hepatoprotective effects both *in vitro* and *in vivo* as evidenced by the increased apoptosis, inhibited ECM accumulation and decreased collagen in HSC-T6 cells. Further, CCE attenuated the TAA-induced changes in various parameters in *in vivo* liver fibrosis rat model. Our findings suggest that CCE may be developed as an effective therapeutic agent against various liver related disorders including liver fibrosis.
